# Dissemination of extended-spectrum beta-lactamase-producing Escherichia coli in poultry in Zimbabwe

**DOI:** 10.1099/mgen.0.001454

**Published:** 2025-07-18

**Authors:** Peter Katsande, Alistair R. Davies, Tom Chisnall, Kudzaishe Vhoko-Tapesana, Sam Willcocks, Chenai S. Majuru, Tendayi Mubau, Richard A. Stabler, Roderick M. Card

**Affiliations:** 1 Department of Veterinary Technical Services, 18A Bevan Building, Liberation Legacy Way, Harare, Zimbabwe; 2 Animal and Plant Health Agency, Woodham Lane, New Haw, Addlestone, UK; 3 Food and Agriculture Organization of the United Nations, Block 1, Tendeseka Office Park, Eastlea, Harare, Zimbabwe; 4 Department of Life Sciences, Brunel University of London, Kingston Lane, Uxbridge UB8 3PH, UK; 5 Department of Infection Biology, The London School of Hygiene and Tropical Medicine, Keppel St, London WC1E 7HT, UK; 6 Masvingo Provincial Veterinary Laboratory, Masvingo, H. Chitepo St, P.O Box 30, Zimbabwe

**Keywords:** *Escherichia coli*, extended-spectrum beta-lactamase (ESBL), plasmid, Zimbabwe

## Abstract

Extended-spectrum beta-lactamase (ESBL)-producing *Escherichia coli* are resistant to the critically important third- and fourth-generation cephalosporin antibiotics and present a risk to animal and human health. In Zimbabwe, there is an evidence gap concerning the prevalence and diversity of ESBL-producing *E. coli* in poultry. In this study, we screened for ESBL-*E. coli* at farms (*n*=50) and markets (*n*=10) using MacConkey agar supplemented with 4 µg ml^−1^ ceftriaxone. ESBL-*E. coli* were detected at every market and at 21 farms, giving a farm-level prevalence of 42%. Seventy isolates were obtained and tested for antimicrobial susceptibility, whilst 69 of these were further analysed by whole-genome sequencing. A total of eight distinct *bla*_CTX-M_ variants were identified, and 69 out of 70 isolates were multidrug-resistant. Genomic analysis revealed evidence for clonal expansion of an ESBL-producing clone and horizontal gene transfer via plasmids being responsible for the dissemination of ESBL-*E. coli*. Geographic Information System mapping was used to visualize the distribution of the ESBL-producing clones. For example, ST1141 isolates were clonal, having a highly conserved core genome, and harboured *bla*_CTX-M-15_ and 11 additional antimicrobial resistance genes on a ~338 kbp IncHI2 plasmid which was not present in other isolates. This clone was present at nine farms. In contrast, a conserved ~93 kbp IncFII plasmid harbouring *bla*_CTX-M-55_ was present in isolates from three different multilocus sequence types obtained from six farms. This study provides insight into the burden and distribution of ESBL-*E. coli* at poultry farms in Zimbabwe and provides molecular genetic evidence for clonal expansion and plasmid transfer as being important mechanisms for the dissemination of ESBL-*E. coli* in this setting. This study underscores the importance of adopting measures, such as prudent antimicrobial use and farm biosecurity, that can limit the development and dissemination of ESBL-producing *E. coli*.

**Impact Statement**

This study provides critical insights into the prevalence, diversity and transmission dynamics of extended-spectrum beta-lactamase (ESBL)-producing *Escherichia coli* in Zimbabwe’s poultry sector. By employing antimicrobial susceptibility testing, genomic sequencing and Geographic Information System mapping, we identified eight distinct *bla*_CTX-M_ variants, all residing in multidrug-resistant isolates. Our findings reveal clonal expansion and horizontal gene transfer as key drivers of ESBL dissemination, with evidence of a dominant ST1141 clone spreading across multiple farms. These results provide fresh insight into the understudied African poultry sector and emphasize the urgent need for targeted interventions, enhanced biosecurity and stricter antibiotic stewardship policies in livestock production. Addressing these gaps through a One Health approach will be essential in mitigating the public health risks associated with antimicrobial resistance and safeguarding both animal and human health.

## Data Summary

The whole-genome sequences were deposited in the National Center for Biotechnology Information National Library of Medicine under BioProject accession number PRJNA1223857. Individual accession numbers are available in Table S1.

## Introduction

The global rise of bacterial antimicrobial resistance (AMR) poses a significant threat to public health, with extended-spectrum beta-lactamase (ESBL)-producing *Escherichia coli* being a leading concern due to their resistance to critically important β-lactam antibiotics, including third-generation cephalosporins [1,2]. *E. coli* and other Enterobacterales resistant to third-generation cephalosporins are classified as critical priority pathogens by the World Health Organization [3]. The spread of ESBL-*E. coli* is facilitated through two primary mechanisms: clonal expansion and plasmid-mediated horizontal gene transfer (HGT). These mechanisms enable the persistence and dissemination of resistance genes within bacterial populations across various hosts and environments [4].

The overuse of antimicrobial drugs in humans and livestock, including poultry, has contributed to the rise and spread of antimicrobial-resistant bacteria, threatening public health, food security and sustainable development. To combat this, the World Health Organization introduced the Global Action Plan on AMR in 2015. In line with this, Zimbabwe implemented its first One Health AMR National Action Plan in 2017, which includes AMR surveillance as a key focus area.

Poultry production systems, particularly in low- and middle-income countries such as Zimbabwe, are increasingly recognized as hotspots for the emergence and spread of ESBL-*E. coli*. The widespread use of antimicrobials in poultry farming for growth promotion and disease prevention [5,6] has contributed to the selection and proliferation of resistant strains [7,8]. This not only poses a risk to animal health and productivity but also facilitates the transmission of resistant bacteria and resistance genes to humans via the food chain, direct contact, or environmental contamination [9].

The broiler sector dominates poultry farming in Zimbabwe, with around 72 million day-old chicks produced each year [10]. In 2022, ~191,813 million tonnes of chicken meat were produced, marking a 34% increase from 2021 (Second Round Crop and Livestock Assessment Report, 2022/2023 season). Notably, 73% of broiler meat production comes from small- and medium-scale producers [11].

ESBL and AmpC-like enzymes represent major bacterial resistance mechanisms, primarily mediated by genes located on plasmids [12,13]. In *E. coli*, resistance is typically developed through mechanisms like efflux pump interference and plasmid-borne resistance genes [14]. Plasmids are thus critical in acquiring and disseminating ESBL genes amongst bacterial populations in humans, animals and the environment, with HGT being the primary driver of antibiotic resistance gene dissemination [15,16]. In Zimbabwe, studies have reported the presence of ESBL-producing *E. coli* in various reservoirs, including people, poultry, domestic pigs, retail meat and wastewater [17,20]. Amongst the most commonly identified resistance genes are *bla*_CTX-M_ and *bla*_TEM_, commonly associated with IncF and IncI plasmid groups [21]. A study on *E. coli* from poultry in Zimbabwe has revealed the presence of Col, IncY, IncI, IncF, IncIH, IncIQ, IncIX and p0111 plasmid types, emphasizing the widespread dissemination of these resistance elements across bacterial populations [19].

Globally, the prevalence of ESBL-producing *E. coli* in food animals has been rising, yet most studies in sub-Saharan Africa have focused on human clinical and community settings [22,23]. In Zimbabwe, little is known about the genetic diversity and mechanisms driving the dissemination of ESBL-producing *E. coli* in poultry. Given the close proximity of animals to humans and their critical role in the food chain, understanding poultry as a potential reservoir for ESBL-producing *E. coli* is crucial for identifying sources of farm-level transmission.

This study aimed to address this evidence gap by investigating the prevalence, genetic diversity and dissemination mechanisms of ESBL-producing *E. coli* in poultry from Masvingo, Zimbabwe.

## Methodology

### Study design

The survey was conducted from June to August 2022, targeting broiler chickens across 50 farms and 10 live bird markets in Masvingo Province, located in south-eastern Zimbabwe. This region includes the districts of Masvingo, Gutu, Zvishavane, Chivi, Zaka, Bikita and Mwenezi. The selection of farms and live bird markets was based on the farmers' voluntary consent to participate in the study, as well as the scale of production. Farms with continuous broiler production were classified as either small- (25–2,000 broiler chickens per batch) or medium- (2,001–10,000 broiler chickens) scale broiler production.

### Sample collection

Samples were collected from chickens at 50 poultry farms and 10 live bird markets. At farms, one pooled sample was collected per poultry house, and in total, 285 houses were sampled. A pooled sample from a farm consisted of three to ten individual cloacal swabs, with the exact number depending on the total number of birds present (typically ranging from 10 to 200 birds). At each market, 12 pooled samples were collected, with each pool composed of five individual cloacal swabs. To create a pool, swabs were placed in one container with Amie’s medium and mixed. In total, 285 pooled samples from poultry farms and 120 pooled samples from markets were collected in this manner. All samples were collected independently from the farms and live bird markets. Collection and transportation of samples adhered to standard guidelines [24]. Each sample was placed in Amie’s transport media, kept at 5 °C±3 °C and delivered to the Central Veterinary Laboratory within 48 h of sample collection for bacteriological examination.

### Isolation and characterization of *E. coli*

Detection of *E. coli* was conducted according to the method published by the Fleming Fund [25]. Briefly, swabs were added to 10 ml of sterile Buffered Peptone Water (BPW) (Oxoid, Basingstoke, Hampshire, UK) and incubated aerobically at 36±2 °C for 16–20 h. The enriched BPW was inoculated onto MacConkey agar (Oxoid, Basingstoke, Hampshire, UK). For ESBL screening, a loopful of the enriched BPW was inoculated onto MacConkey agar supplemented with 4 µg ml^−1^ of ceftriaxone and streaked to obtain single colonies [26]. The plates were incubated aerobically for 18–22 h at 37±0.5 °C. Typical *E. coli* colonies (pink, indole positive, urease negative) from the plates were sub-cultured to blood agar (Oxoid, Basingstoke, Hampshire, UK) and incubated aerobically for 18–22 h at 37±0.5 °C. *E. coli* confirmation was performed with the API-20E biochemical test (API-20, bioMérieux, UK and Ireland).

### ESBL confirmation

Phenotypic confirmation of ESBL on the *E. coli* isolates was performed using the combined disc test [27]. Mueller–Hinton agar plates were inoculated with bacterial suspension adjusted to 0.5 McFarland standard. Antibiotic discs containing cefotaxime (30 µg) and ceftazidime (30 µg), along with their respective combinations with clavulanic acid (10 µg) (Oxoid, Thermo Fisher Scientific), were placed on each plate with adequate spacing (≥20 mm). The plate was incubated at 36±1 °C for 18–24 h. After incubation, the zone diameters around the discs are measured. Quality control for each batch of cephalosporin-containing MacConkey agar plates was conducted using *E. coli* ATCC 25922 and a *bla*CTX-M-15-positive *E. coli* isolate. The confirmed ESBL-producing *E. coli* isolates (zone difference ≥5 mm) were stored long-term at −80 °C in 30% glycerol (v/v) in tryptic soy broth.

### Antimicrobial susceptibility testing

Antimicrobial susceptibility testing was performed on isolates by broth microdilution for MIC determination using commercial plates (Sensititre^™^ EU Surveillance *Salmonella/E. coli* EUVSEC3 plate, Thermo Fisher Scientific, 2021), according to the manufacturer’s instructions. Briefly, a suspension of each isolate was adjusted to a density of 0.5 McFarland in 5 ml of demineralized water, and then, 10 µl of the suspension was transferred to 11 ml of Mueller–Hinton broth to obtain a target inoculum density of between 1×10⁵ and 1×10⁶ c.f.u. ml^−1^. Fifty microlitres were dispensed into each well of the microtitre plate using a Sensititre AIM Automated Inoculation Delivery System (ThermoFisher Scientific) and incubated aerobically at 35–37 °C for 18–22 h. Antimicrobials tested comprised amikacin, ampicillin, azithromycin, cefotaxime, ceftazidime, chloramphenicol, ciprofloxacin, colistin, gentamicin, meropenem, nalidixic acid, sulphamethoxazole, tetracycline, tigecycline and trimethoprim. *E. coli* NCTC 12241 (ATCC 25922) was used as the control strain. Susceptibility was assessed using EUCAST ECOFF values (accessed on 21 November 2022) [28], except for sulphamethoxazole for which the interpretative criteria proposed by the European Food Safety Authority [29] were employed as an ECOFF value was not available [30]. ECOFFs distinguish micro-organisms without (wild type) and with phenotypically detectable acquired resistance mechanisms (non-wild-type) to the antimicrobial in question (https://mic.eucast.org/). In this paper, the use of the term ‘resistance’, such as multidrug resistance (MDR) and phenotypic resistance, refers to the non-wild-type phenotype, which is not necessarily synonymous with clinical resistance [28,30]. Isolates resistant to third-generation cephalosporins (cefotaxime MIC ≥0.5 mg l^−1^ and/or ceftazidime MIC ≥1 mg l^−1^) were additionally tested on the EUVSEC2 microplate (Sensititre^®^, Trek Diagnostic Systems, East Grinstead, UK) to determine the presumptive phenotype of ESBL and AmpC, and producers [29]. Antibiotics included in the EUVSEC2 plate were cefepime, cefotaxime, cefotaxime and clavulanic acid, cefoxitin, ceftazidime, ceftazidime and clavulanic acid, ertapenem, imipenem, meropenem and temocillin. MDR was defined as non-susceptibility to three or more antimicrobial classes [30].

### Whole-genome sequencing and analysis

Genomic DNA was prepared from overnight Luria–Bertani broth cultures with the MagMAX^™^ CORE extraction kit (Thermo Fisher Scientific, Basingstoke, UK) using the semi-automated KingFisher Flex system (Thermo Fisher Scientific, Basingstoke, UK) according to the manufacturer’s instructions. Extracted DNA was processed for whole-genome sequencing (WGS) using the NextSeq® 500/550 Mid Output Kit v2.5, using NextSeq sequencing reagents. The resulting raw sequences were analysed with the Nullarbor 2 pipeline [31], using, as reference, the published genome *E. coli* K12 (accession number U00096.2). SPAdes was used for genome assembly (version 3.14.1 [32]) and Prokka for annotation (version 1.14.6 [33]). One genome did not pass quality control (contigs>2,000 and N50 <3,500 bp) and was removed from further analysis. The presence of genes and point mutations conferring AMR and disinfectant tolerance was assessed using AMRFinderPlus [34]. Genes encoding heavy metal tolerance and plasmid incompatibility types were identified using APHA Seqfinder (https://github.com/APHA-AMR-VIR/APHASeqFinder). The MLST data were determined with MLST software (version 2.19.0 [35]) using the PubMLST database [36]. Core genome SNPs were generated using SNIPPY [37]. Phylogenetic trees with 200 bootstraps were built using RAX-ML [38] from the core genome SNPs and annotated using iTOLv5 [39]. DNA extracts were also used to screen for the presence of the Shiga toxin-encoding *stx* gene by PCR [40].

A subset of 24 isolates was selected based on sequence type (ST) and *bla*_CTX-M_ gene for long-read sequencing using Oxford Nanopore Technologies. DNA was extracted using the GenFind V3 extraction kit (Beckman Coulter) according to the manufacturer’s instructions. Sample preparation was carried out using the SQK-RBK114.24 Rapid Barcoding Kit (ONT) according to the manufacturer’s instructions. Samples were then run on MinION and MinION flow cell R10.4.1 for 72 h. Hybrid assemblies were created using long- and short-read sequences by Unicycler v2.0 [41] to generate closed (fully circularized) plasmids. StarAMR was used to map AMR resistance genes to plasmids [42]. BLASTn [43] and MOB-suite [44] were used to identify similar published plasmids from the NCBI public database. MOB-recon was used to reconstruct individual plasmid sequences from short-read genome assemblies using the clustered plasmid reference databases provided by MOB-cluster [44]. BRIG [45] was used to generate an image that compared published plasmids with those identified in this study. The CARD AMR database [46] and Bakta [47] were used for image annotation.

The whole-genome sequences were deposited in the NCBI SRA under BioProject accession number PRJNA1223857.

## Results and discussion

### High prevalence of ESBL-*E. coli* at poultry farms and markets

A total of 405 samples were collected from 50 farms (285 pooled samples) and 10 live bird markets (120 pooled samples). The overall *E. coli* positivity rates were 95.4% (272 out of 285) for farms and 93.3% (112 out of 120) for live bird markets. Seventy *E. coli* isolates were identified as ESBL producers, giving an overall occurrence of 18.2% (70 out of 384), 51 isolates (51 out of 285, 17.9%) from farms and 19 isolates (19 out of 120, 15.8%) from live bird markets. ESBL-*E. coli* were isolated from samples collected at all ten markets and from 21 of 50 farms, giving a farm-level prevalence of 42% (Table S1, available in the online Supplementary Material). This compares to a prevalence of 31.4% reported for Nigerian poultry farms [48], 56.2% for backyard chickens in Ghana [49] and 26.8% for broilers in the European Union [50], as exemplars. In 2023, a systematic review of global reports was published that described the pooled prevalence of ESBL-producing *E. coli* in animals as 33.5% [51]. By establishing the prevalence of ESBL-producing *E. coli* poultry in Zimbabwe, we help address an evidence gap identified in a 2023 review which highlighted the absence of such studies from Central and Southern Africa [52]. One previous study in Zimbabwe has described ESBL-producing *E. coli* in poultry [19], but this was focussed on just two farms and additional convenience samples from backyard chickens; ESBL-producing *E. coli* were detected on both farms.

The detection of ESBL-producing *E. coli* at all ten markets most likely reflects an aggregation effect as markets will source poultry from many different farms. Furthermore, it highlights a potential food safety risk, whereby consumers may purchase poultry products contaminated with ESBL-producing *E. coli*. In this study, however, we examined birds and not poultry products such as meat following slaughter at the market. Zimbabwe has legislation and a regulatory framework to support food safety, although challenges and non-compliances in implementation have been reported [53,54], heightening risks to consumers.

### The ESBL phenotype was conferred by *bla*_CTX-M_ genes

All 70 ESBL*-E. coli* isolates were resistant to ampicillin, cefepime and cefotaxime (Tables 1 and S1). They were verified as ESBL producers through synergy with cefotaxime and clavulanic acid and showed a corresponding MIC ratio ≥8 with cefotaxime in accordance with EUCAST guidelines.

**Table 1.** Occurrence of resistance in ESBL-producing *E. coli*

Antimicrobial class*	Antibiotic	No. of resistant isolates	% resistant
Penicillins (aminopenicillins)	Ampicillin	70	100%
Cephalosporins (third and fourth generation)	Cefepime	70	100%
	Cefotaxime	70	100%
	Cefotaxime and clavulanic acid	0	0%
	Ceftazidime	38	54%
	Ceftazidime and clavulanic acid	0	0%
Cephalosporins (first and second generation)	Cefoxitin	0	0%
Carbapenems	Ertapenem	0	0%
	Imipenem	0	0%
	Meropenem	0	0%
Penicillins (aminopenicillins)	Temocillin	0	0%
Aminoglycosides	Amikacin	0	0%
	Gentamicin	17	24%
Amphenicols	Chloramphenicol	62	89%
Macrolides	Azithromycin	6	9%
Polymyxins	Colistin	0	0%
Quinolones	Ciprofloxacin	67	96%
	Nalidixic acid	67	96%
Sulphonamides	Sulphamethoxazole	69	99%
Dihydrofolate reductase inhibitors	Trimethoprim	40	57%
Tetracyclines	Tetracycline	49	70%
Glycylcyclines	Tigecycline	0	0%
Multidrug-resistant		69	99%

*https://www.who.int/news/item/08-02-2024-who-medically-important-antimicrobial-list-2024.

**Fig. 1.**
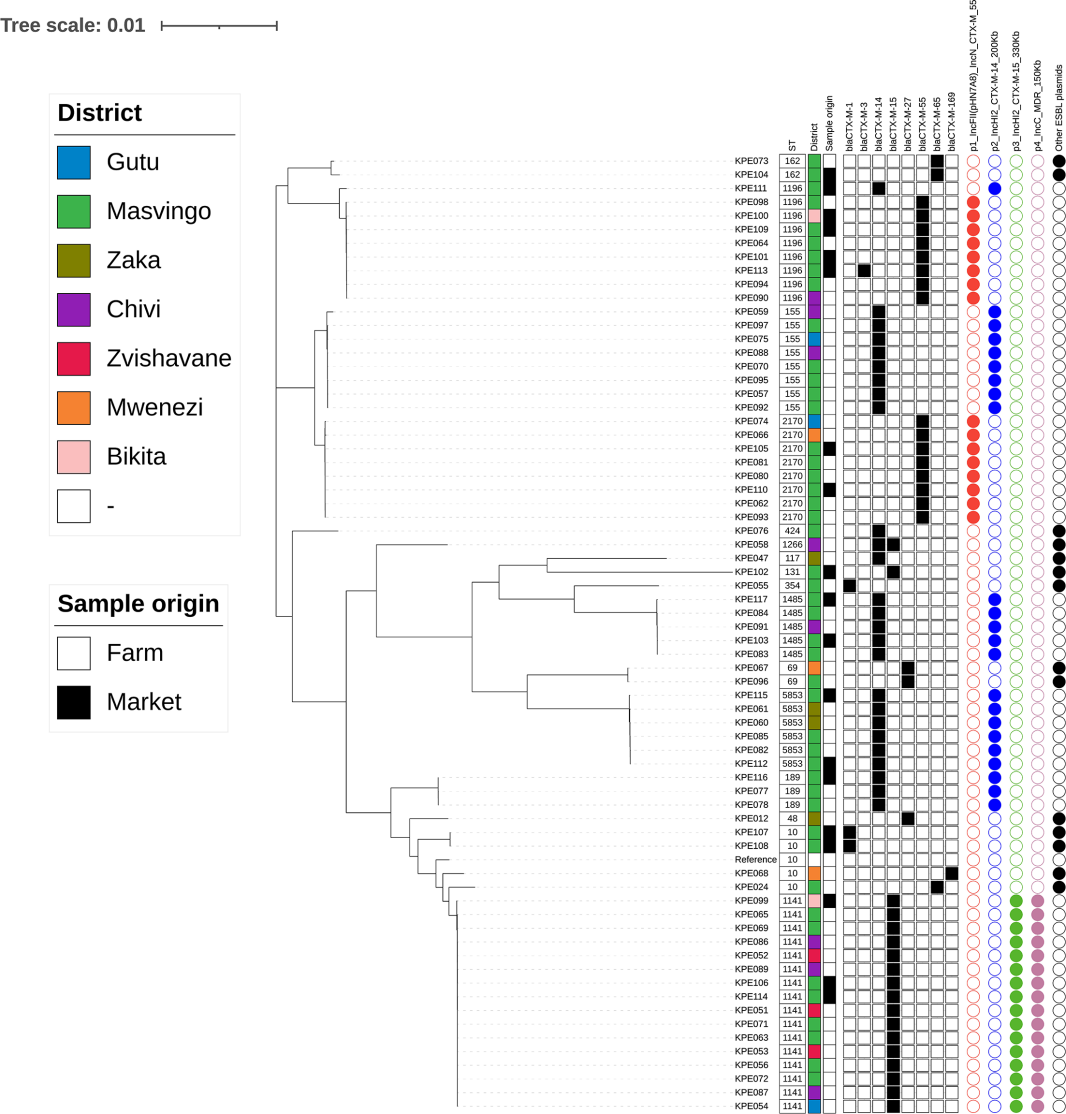
Maximum-likelihood phylogenetic tree generated from core genome SNPs of ESBL-producing *E. coli* obtained from poultry in Zimbabwe. The ST, district and sample source (market or farm) are presented. The presence of *bla*_CTX-M_ variants is indicated by a black box. Plasmids described in the text are indicated by red, blue, green and purple circles. Other plasmids are indicated by a black circle. The reference strain was *E. coli* K12 (accession number U00096.2).

The whole-genome sequence was analysed for 69 isolates (isolate KPE079 did not pass WGS quality control and was excluded from further analysis). The ESBL phenotype was associated with the presence of *bla*_CTX-M_ genes in every isolate, and eight different *bla*_CTX-M_ variants were present in the 69 isolates (Fig. 1): *bla*_CTX-M-1_ [3], *bla*_CTX-M-3_ [1], *bla*_CTX-M-14_ [26], *bla*_CTX-M-15_ [18], *bla*_CTX-M-27_ [3], *bla*_CTX-M-55_ [16], *bla*_CTX-M-65_ [3] and *bla*_CTX-M-169_ [1] (Fig. 1 and Table S1). Two isolates harboured two *bla*_CTX-M_ genes: KPE058 (*bla*_CTX-M-14_ and *bla*_CTX-M-15_) and KPE113 (*bla*_CTX-M-3_ and *bla*_CTX-M-55_); all other isolates had a single *bla*_CTX-M_ gene. In Zimbabwe, *bla*_CTX-M-1_, *bla*_CTX-M-65_ and *bla*_CTX-M-169_ have not been identified previously, and *bla*_CTX-M-3_ and *bla*_CTX-M-27_ have been reported only in isolates from humans [18,19].

### Extensive genetic diversity in ESBL-producing *E. coli* is revealed

WGS also revealed considerable genetic diversity in the genomes of the 69 bacterial isolates, with 17 different MLST STs identified. The core-genome SNP-based phylogenetic tree shows the clustering of isolates into clades according to ST (Fig. 1). The most frequently detected STs were ST1141 (*n*=16), ST1196 (*n*=9), ST155 (*n*=8) and ST2170 (*n*=8) (Fig. 1 and Table S1). Nine STs were detected only once, of which one was ST131. Eight STs have been reported previously in ESBL-producing *E. coli* in Zimbabwe: ST69, ST117, ST131, ST155 and ST354 from humans [18] and ST10, ST48, ST117, ST155 and ST1196 from poultry [19].

**Fig. 2.**
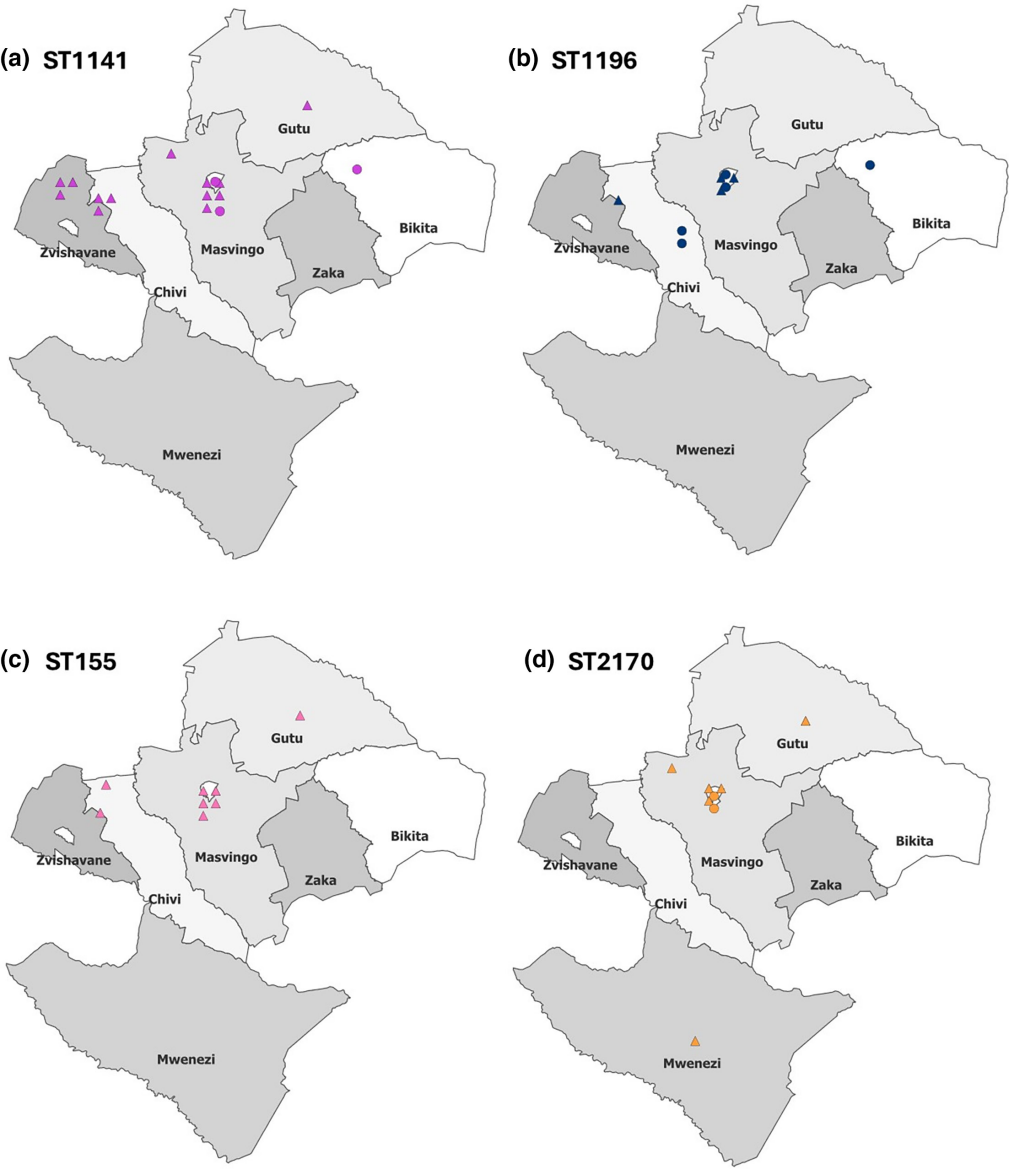
Distribution of ESBL-*E. coli* STs in Masvingo region. *E. coli* were isolated from farms (triangle) or markets (circles). Multiple isolates from a single location were displayed in grid form around a centre point (not marked). The four most common STs are displayed: (a) ST1141, (**b**) ST1196, (**c**) ST155 and (**d**) ST2170.

Many STs showed a wide geographical distribution as they were present in multiple districts and detected at both farms and markets (Fig. 2 and Table S1). For example, ST1141 was detected in five districts, and ST1196, ST155 and ST2170 were each detected in three districts (Fig. 2 and Table S1). At least two different STs were present in each district, with Masvingo, the most heavily sampled district, having 12 different STs. Some STs were present at multiple farms and markets; for example, ST1141 was detected on nine farms and at three markets, ST1196 at three farms and four markets, ST155 at six farms and ST2170 at five farms and two markets (Table S1). Multiple STs were frequently detected at the same sampling site; 6 of the 10 markets had 2 or 3 STs present, and 13 of 21 farms possessed 2–4 different STs (Table S1). This indicates, together with similar findings reported by Takawira *et al*. [19], that multiple lineages of ESBL-producing *E. coli* co-exist at the same farm in Zimbabwe.

### Dissemination mechanisms of the ESBL phenotype

We observed a correlation between ST and *bla*_CTX-M_ gene type and explored this further using long-read sequencing and hybrid genome assemblies for 24 isolates (at least one from each ST and *bla*_CTX-M_ gene type combination) which enabled assessment for the presence of plasmids and the location of the *bla*_CTX-M_ genes. A total of 12 different plasmids, from 7 different incompatibility types, harbouring a *bla*_CTX-M_ gene were identified (Fig. 1 and Table S2), indicating a considerable diversity in mobile elements. For isolates KPE058 and KPE113 (each harbouring two different *bla*_CTX-M_ genes), one *bla*_CTX-M_ gene was present in the bacterial chromosome (associated with IS6 family insertion sequence elements), and one resided on a plasmid (Tables S1 and S2). The *bla*_CTX-M-65_ gene in isolate KPE104 was located on the chromosome and associated with an IS3 family insertion sequence element. For all other isolates, the *bla*_CTX-M_ gene was located on a plasmid. The widespread diversity and distribution across the study farms and markets of ESBL-producing *E. coli* and the plasmids that they harboured were notable and provided some insight into potential dissemination mechanisms.

**Fig. 3.**
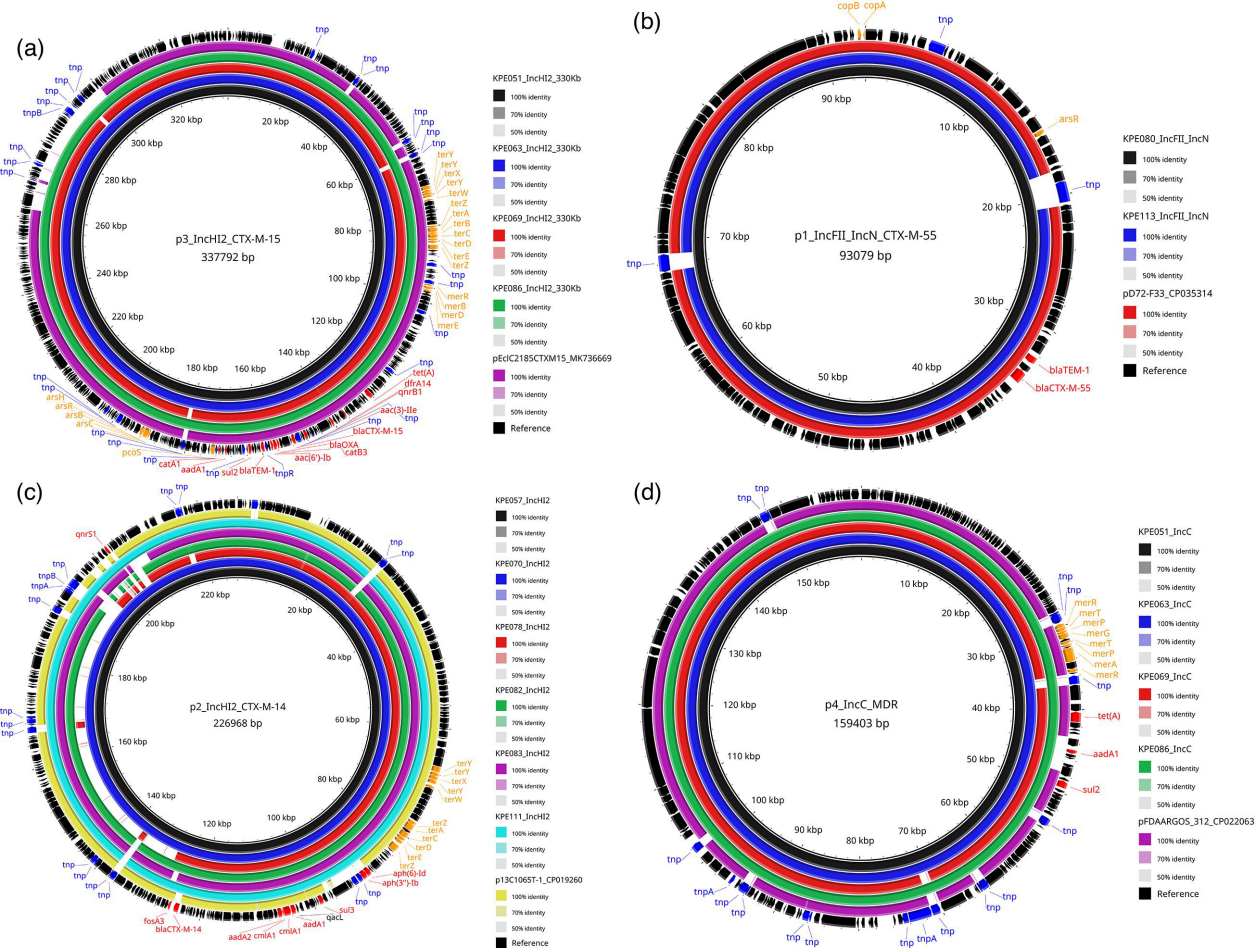
Comparative analysis of four plasmids harbouring AMR genes. The shade of colour indicates the blast percentage identity. The arrows around the map indicate deduced CDSs and their orientation. AMR genes are labelled in red, heavy metal tolerance genes in orange and transposons in blue. Images were generated using BRIG. (a) Plasmid p3_IncHI2_CTX-M-15 from four isolates in this study (black, blue, red and green rings) and the most similar Mash neighbour identified using MOB-suite (purple ring). (b) Plasmid p1_IncFII_IncN from two isolates in this study (black and blue rings) and the most similar Mash neighbour identified using MOB-suite (red ring). (c) Plasmid p2_IncHI2_CTX-M-14 from six isolates in this study (black, blue, red, green, purple and cyan rings) and the most similar Mash neighbour identified using MOB-suite (yellow ring). (d) p4_IncC_MDR from four isolates in this study (black, blue, red and green rings) and the most similar Mash neighbour identified using MOB-suite (purple ring).

Evidence for clonal expansion was provided by the ST1141 isolates which were highly conserved in their core genome, with a median difference of <20 SNPs and many with <15 SNP difference (Table S3), indicating that they were clones [55]. These 16 isolates harboured a highly conserved ~338 kbp IncHI2 plasmid with a 99.99% nucleotide identity, carrying *bla*_CTX-M-15_, 11 additional AMR genes, genes conferring tolerance to heavy metals (*ars*, *mer*, *pco* and *ter*) and multiple transposon genes (*tnp*) (Fig. 3a). The plasmid-encoded AMR genes are able to confer resistance to eight antimicrobial classes and can be considered an MDR plasmid. This MDR plasmid was not present in other isolates. The ST1141 clone was the most widely distributed ST (detected at nine farms and three markets in five districts) and may represent a locally successful clone present in poultry production in Zimbabwe. ST1141 was not detected in poultry or humans from previous studies in Zimbabwe [18,19] but has been reported in cattle in China, where it was associated with *bla*_CTX-M-17_ [56].

Plasmids are recognized as important vehicles for the dissemination of AMR genes between bacteria that are not related by direct descent [13]. We identified two conserved plasmids that provide exemplars of plasmid-mediated dissemination of the ESBL-producing phenotype in Zimbabwe: a ~93 kbp IncFII/IncN mosaic plasmid harbouring *bla*_CTX-M-55_ (Fig. 3b) and a ~227 kbp IncHI2 plasmid harbouring *bla*_CTX-M-14_ (Fig. 3c). These two plasmids were each present in isolates from various STs and consequently resided in several clades of the phylogenetic tree (Fig. 1).

The ~93 kbp IncFII/IncN plasmid was identified in ST1196 and ST2170 isolates and had 99.96% nucleotide identity between the two long-read exemplars (one from each ST). The plasmid carried *bla*_CTX-M-55_, *bla*_TEM_, copper (*copA*, *copB*) and arsenic (*arsR*) resistance genes, and three transposons (*tnp*) (Figs 1 and 3b). The plasmid was identified in isolates from six different farms. ST1196 has been described as an increasingly prevalent and international high-risk ST, associated with resistance to colistin, third-generation cephalosporins, carbapenems and/or aminoglycosides [57,58]. It has been primarily isolated from poultry but has also been identified in water treatment plants, fish and human clinical samples [57,58]. An ESBL-producing ST1196 harbouring the *bla*_CTX-M-79_ gene has been previously described in Zimbabwe poultry [19]. ST2170 has been isolated from poultry meat in Korea [59] and pigs in Vietnam (associated with *bla*_CTX-M-55_) [60] but, overall, has been rarely reported with only 21 examples in Enterobase (accessed on 21 January 2025) [61].

The ~227 kbp IncHI2 plasmid was present in isolates from five different STs: ST155, ST189, ST1196, ST1485 and ST5853 (Fig. 1). It had a nucleotide identity of >99.98% and a coverage between 69 and 100% for the five long-read exemplars. This plasmid carried *bla*_CTX-M-14_, up to nine additional AMR genes, the tellurite resistance gene (*ter*) operon, a disinfectant resistance gene (*qacL*) and multiple transposon genes (*tnp*) (Fig. 3c and Table S2). This MDR plasmid was identified in isolates from nine different farms. We observed that this plasmid, although retaining a conserved core, differed in its gene complement between isolates (Fig. 3c and Table S2). The plasticity of plasmids and their capacity to gain, lose and rearrange genes is widely recognized [62,63]. The ST155 *E. coli* lineage has been described as a potential food-borne pathogen as it has been associated with disease in both poultry and humans [64]. ST1485 is disseminated worldwide in humans, animals and the environment and has been associated with *bla*_CTX-M-14_ [65]. Interestingly, although eight ST1196 isolates had this ~227 kbp IncHI2 plasmid, one ST1196 isolate possessed the ~93 kbp IncFII plasmid. This highlights the importance of plasmids in the dissemination of AMR determinants.

### High occurrence of MDR

The most frequently observed additional resistances in the ESBL-producing isolates were towards sulphamethoxazole (69 out of 70; 99%), the quinolones ciprofloxacin and nalidixic acid (67 out of 70; 96%) and chloramphenicol (62 out of 70; 89%) (Tables 1 and S1). Resistance to tetracycline, trimethoprim or ceftazidime was observed in over half of the isolates (Table 1). Sixty-nine (99%) isolates were multidrug-resistant, being non-susceptible to three or more antimicrobial classes. ESBL-producing *E. coli* with MDR and a high prevalence of resistance to these antimicrobials is often reported, including in Zimbabwe [18,19]. All isolates were susceptible to amikacin, carbapenems (ertapenem, imipenem and meropenem), cefoxitin, colistin, temocillin and tigecycline.

Resistance to these antibiotics was associated with the following genes: azithromycin [*mph(A*)], chloramphenicol (*catA1*, *catB3* or *floR*), gentamicin [*aac (3)-IIe* and *aac(6')-Ib-cr5*], sulphamethoxazole (*sul*), tetracycline resistance [*tet*(A)] and trimethoprim (*dfrA*) (Table S1). All isolates resistant to ciprofloxacin and nalidixic acid possessed a mutation in the *gyrA* gene that causes the S83L amino acid substitution associated with quinoline resistance [66]. Many isolates harboured additional quinolone resistance determinants such as mutations in *parC*, *aac(6')-Ib-cr5* and/or a *qnr* gene (Table S1). There was excellent correspondence (99%) between the AMR phenotype (as determined by MIC) and the AMR genotype (as determined by WGS) (Table S1). All isolates possessed additional AMR genes and/or mutations which confer resistance to antimicrobials not in the panel tested by broth microdilution (Table S1).

Finally, we identified additional plasmids which did not carry a *bla*_CTX-M_ gene but did harbour other AMR genes. As an exemplar, we identified a highly conserved (99.9% nucleotide identity) ~159 kbp IncC plasmid with three AMR genes [*sul2*, *aadA1* and *tet*(A)) that was present in the ST1141 isolates (Figs 1 and 3d).

Isolate KPE102 was ST131 and was positive for *pap* operon gene *papA-K* (data not shown), which encodes factors that mediate adhesion to uroepithelial cells and are therefore important in urinary tract infections [67]. *E. coli* ST131 is a globally distributed, multidrug-resistant extraintestinal pathogenic *E. coli* lineage associated with severe human infections in community and hospital settings [4]. ST131 *E. coli* can colonize the gastrointestinal tracts of animals and have been previously isolated from poultry worldwide, including from Asia, Europe, North America and Oceania [68]. In Zimbabwe, ST131 was a predominant ESBL-*E. coli* lineage in a study examining human urinary tract infections [18]. All isolates in this study were negative for the Shiga toxin-encoding *stx* gene. Genes associated with increased tolerance to disinfectants (*qacEdelta1* and *qacL*) were detected in 41 isolates (59%) (Table S1). Disinfectant and heavy metal tolerance genes were often located on the plasmids encoding a *bla*_CTX-M_ gene (Fig. 3 and Table S2), and there is growing evidence to show that disinfectants and heavy metals can co-select for AMR in bacteria [69].

## Conclusions

Poultry farming plays a significant role in enhancing food security in Zimbabwe, serving as an essential source of protein and a primary income stream for many households, amid the country’s high unemployment rate. In the Zimbabwe broiler sector, non-prudent practices and limited input from professional veterinary services for disease diagnosis and treatment are common [5] and can lead to increased risk for the development and dissemination of ESBL-producing *E. coli* [70]. Moreover, the presence of ESBL-*E. coli* in food-producing animals has emerged as a significant global challenge, impacting both public health and food security [71].

In this study, we have determined a farm-level prevalence of ESBL-*E. coli* that is high compared to many other settings, emphasizing a potential risk to the health of people and livestock. There is considerable diversity in ESBL-*E. coli* in Zimbabwe, with many STs having been detected in both people and poultry. The association between HIV and ESBL-producing *E. coli* in patients with urinary tract infections in Zimbabwe [20] highlights these public health concerns. All ESBL isolates (except one) were MDR and quinolone-resistant, further limiting treatment options should they cause an infection in people or livestock.

Our study provides the first insight into the broader burden and distribution of ESBL-*E. coli* in small- and medium-scale poultry farms in Zimbabwe. We provide molecular genetic evidence for clonal expansion and plasmid transfer for the dissemination of ESBL-*E. coli* in the poultry production system in Zimbabwe. Future studies could be designed to identify possible sources and dissemination routes of ESBL-producing isolates, such as through the breeding pyramid, feed, wildlife and inadequate biosecurity practices on farms. In this manner, context-relevant interventions and mitigations can be designed and implemented to reduce the risk of development and dissemination of AMR at poultry farms in Zimbabwe.

## Supplementary material

10.1099/mgen.0.001454 Uncited Supplementary Material 1.

## Abbreviations

AMR: antimicrobial resistance
BPW: Buffered Peptone Water
ESBL: extended-spectrum beta-lactamase
HGT: horizontal gene transfer
MDR: multidrug resistance
MIC: Minimum Inhibitory Concentration
ST: sequence type
WGS: whole-genome sequencing
